# The Additional Information of Bitewing Radiographs in the Detection of Established or Severe Dentinal Decay in 14-Year Olds: A Cross-Sectional Study in Low-Caries Population

**DOI:** 10.1155/2014/175358

**Published:** 2014-01-16

**Authors:** Aija-Maaria Hietala-Lenkkeri, Mimmi Tolvanen, Pentti Alanen, Kaisu Pienihäkkinen

**Affiliations:** ^1^Carea Joint Municipal Authority for Medical and Social Services in Kymenlaakso, Kuusankoski, 06100 Porvoo, Finland; ^2^Department of Community Dentistry, Institute of Dentistry, University of Turku, 20520 Turku, Finland

## Abstract

*Aim*. To reveal the additional value of radiographic bitewings (BW) in detection of caries and in comparing the occurrence of clinically undetected severe decay between 14-year olds with and without clinically observed dentinal caries in a low-caries prevalence population. *Design*. The cross-sectional study used 363 pairs of radiographs read by one examiner without knowledge of the clinical findings. The yield was analyzed on a tooth surface level by cross tabulating the clinical and radiographic information and on an individual level by counting the number of yield surfaces for all subjects. Mann-Whitney *U* test was used. *Results*. On a tooth surface level, the contribution of BW was the greatest on the occlusal surfaces of the first molars, where established or severe dentinal decay was registered in BW in 11% of clinically sound surfaces and in 40% of established cavitated enamel lesions. On an individual level, 53% of subjects benefited from BW. The subjects clinically DMFS > 0 benefited more than the clinically DMFS = 0 subjects (*P* = .004), nearly 60% in relation to 47%, respectively. *Conclusions*. In a low-caries prevalence population a remarkable portion of both clinically DMFS = 0 and DMFS > 0 14-year olds benefit from BW examination. Most of the benefit is obtained on the occlusal surfaces of the first and the second permanent molars.

## 1. Introduction

In Sweden and Norway, for example, bitewings have been routinely—in more than 90% of subjects over 11 years of age—obtained in connection with the annual clinical dental examination or every two years [1]. In Finland, however, the common practice in obtaining the radiographs is much more prudent and fiber-optic transillumination is routinely used in the detection of approximal caries. The Finnish Current Care Guidelines of caries [2] recommend that the radiographs should be taken if dentine caries is detected in a clinical examination.

The relevance of bitewing examination (BW) in the detection of caries has been studied in several, predominantly histological studies on extracted teeth. As in other Western countries, caries prevalence declined during recent decades in Finland. The clinical detection of the lesions, progressing slowly and not showing cavitation until a later stage of the disease progression than earlier, is difficult. In a systematic review of the Swedish Council on Technology Assessment in Health Care (SBU) it was concluded that visual/tactile examination has limited reliability in the detection of enamel and early dentine caries in occlusal surfaces of the posterior teeth. This method was not considered sufficient in establishing the presence of dentine caries in the approximal surfaces of the posterior teeth, either. The authors also stated that the radiographic examination has a high specificity but low sensitivity in the detection of dentine caries of approximal surfaces. On the occlusal surfaces, the radiographic examination may lead to a moderate overregistration of dentine caries and the probability of a false-positive registration increases significantly with decreasing prevalence of caries. The combination of visual/tactile and radiographic examination increases the likelihood of a positive diagnosis being correct when compared with either method alone [1].

The lesion slowly progressing under the sound-looking enamel may not be perceptible to the clinical diagnostic techniques [3–5]. In low-caries prevalence populations the prevalence of these hidden caries lesions has increased, possibly as a result of the intensive use of fluoride [6–8]. On the other hand, it has also been observed that fluoride may reduce the occurrence of hidden lesions [9]. This change in the morphology of many caries lesions has resulted in a reduction in the sensitivity of the clinical registration of caries [10]. In addition, the predilection site of the lesions has altered and a higher proportion of lesions is now seen on the occlusal surfaces [4].

The additional value of bitewing radiographs in the detection of approximal caries is widely recognized [11–13]. However, opinions on their significance in the detection of occlusal caries are more controversial [13–15]. Further, the contribution of the radiographs has been studied in several studies with subjects with primary dentition [16–18], possibly not revealing anything about the yield of BW screening for young adolescents with newly erupted permanent teeth.

Thus, the hypothesis of the study was that in low-caries conditions BW radiographic examination is needed in addition to a clinical observation in caries detection. A null hypothesis was that the hidden obvious dentinal decay calling for restorative treatment is as common in subjects with clinically detected dentinal caries or fillings as in subjects with no experience of obvious caries or fillings.

Further, the aims of the study were to investigate the eventual additional value of bitewing examination in the detection of occlusal and approximal caries and to compare the occurrence of the clinically undetected established or severe dentinal caries between adolescents with and those without clinically observed dentinal caries.

## 2. Material and Methods

### 2.1. Sample

The clinical and radiographic data of the present cross-sectional study came from a 4-year cluster-randomized, double-blinded clinical trial investigating the caries preventive effect of polyol lozenges relative to a control group (*n* = 496) [19]. All subjects from the fourth grade in 21 schools in the city of Kotka in 1999 were eligible to participate in the original trial. At the final clinical examination of the trial posterior bitewing radiographs were obtained (*n* = 520). The analyses used data from 363 subjects (198 girls and 165 boys) with a mean age of 14 years. The average DMFT of the 12-year olds of Kotka was approximately 0.8 (the annual report of Kotka Health Center) in comparison to a Finnish average of 1.2 [20].

### 2.2. Clinical Examination

Prior to the commencement of the study, one of the authors (PA), experienced in epidemiological trials, instructed the single dentist who carried out the clinical examinations (AHL). The emphasis was on avoidance of overregistration of caries. The examiner was not familiar with any of the subjects, neither did she belong to the ordinary health center personnel treating the adolescents. For practical reasons, the clinical dental examination of nine subjects was done by their ordinary dentist. Also in these cases, the radiographs were obtained during the same visit for the clinical dental examination. Details of the clinical examination as well as the criteria used to assess caries have been reported earlier [19]. An air booster, dental explorer, fiber optics, and a mouth mirror were used in the examination, which was performed at the local health center dentists' offices in a dental chair with an ordinary working light after the subject had brushed his or her teeth with fluoride toothpaste. Caries was registered using a modified WHO procedure [21]. These scorings were further recoded and placed in up-to-date perspective using codes in line with those of the International Caries Detection and Assessment System (ICDAS) [22].

WHO codes zero and one, corresponding codes zero to two in the ICDAS classification (ICDAS 0–2), were considered one category. An enamel lesion with a clinically detectable loss of substance but with no obvious spread in the dentine, that is, ICDAS 3, was regarded as established enamel decay. Dentine caries obviously spreading in the dentine, ICDAS 4–6, was considered established and severe dentinal decay calling for filling.

Lesions and restorations were registered at the surface level. There were no teeth extracted due to caries. Caries and eruption status of all permanent teeth were registered, even though not all teeth or surfaces were included in the present analyses. The DMFS was calculated on an individual level, and it included clinically registered ICDAS 4–6 codes. The subjects were categorized to those having DMFS > 0 and those having DMFS = 0, that is, subjects with and without clinically observed dentinal caries, respectively.

### 2.3. Radiographic Examination

One pair of posterior bitewing radiographs was obtained using Kodak Ultra-Speed D safety one film and one of the six X-ray units of the Kotka health center. The units used were Sirona (60 kV, 7 mA, and 0.50 s.), Planmeca (63 kV, 8 mA, and 0.25 s.), Siemens Heliodent (60 kV, 7 mA, and 1.20 s.), Trophex (60 kV, 12 mA, and 0.50 s.), Heliodent DS (60 kV, 7 mA, and 0.60 s.), and Sirona (60 kV, 7 mA, and 0.50 s.). The subject wore a leaded safety collar. Kwik-Bite film holders (Hawes-Neos Dental, Bióggio, Switzerland) and a standardized method in the focusing of the beam were applied. This included that a shaft in the holder guided the focusing. Processing of the films was performed manually by one experienced dental nurse.

The radiographs were ID-coded, and no name of the subject was used. The radiographs were read by a single examiner (AHL) without knowledge of the clinical recordings in a dark room using an illuminating view-box and 1.5-fold magnification. The radiographic recordings were added to the file on the clinical registrations. That is, the radiographic assessment could not decrease the number of decayed surfaces registered in a clinical examination.

The occlusal surfaces of the 1st and the 2nd permanent molars, the mesial surfaces of the 2nd premolars and the 1st and the 2nd molars, and the distal surfaces of the 1st and the 2nd premolars and the 1st molars were examined and included in the analyses.

In the radiographs, caries was registered using a modification of the Mejáre [23] scoring system, in which an approximal caries lesion is given a value between one and five according to the depth of the lesion. As an exception to this system, radiolucency with obvious spread in the dentine (Mejáre's classes four and five) even on an occlusal surface was also registered. The bitewing registration did not include an assessment of early dentinal lesions on the occlusal surfaces of the molars.

Radiolucency in the outer half of the enamel or deeper but with no spread in the dentine was recorded as an established enamel lesion. Radiolucency with an obvious spread in the dentine, that is, in the outer half and deeper, was recorded as an established or severe dentine decay. Secondary caries and a filling with a new primary dentine caries lesion on the same surface were registered as established and severe dentine decay and a filling with a new primary enamel caries lesion on the same surface was registered as a filling.

Of the total of 5 808 teeth (11 616 surfaces) potentially available for recordings in 363 subjects, 199 (3.4%) teeth, that is, 426 (3.7%) surfaces, were excluded from the radiographic examination. The reasons for this were as follows: a tooth was unerupted (*n* = 94), it was congenitally missing (*n* = 42), or it had been extracted for an orthodontic reason (*n* = 63). A surface could also be excluded because of being “unreadable” (*n* = 94). This meant the presence of an orthodontic appliance, a restorative treatment performed due to hypo-mineralization (a clinical registration), or a technical reason (wrong positioning of the film, overlapping, and under- or overexposure).

### 2.4. Yield from the Radiographs

Detection of established or severe dentinal decay (ICDAS 4–6) on a surface clinically registered ICDAS 0–2 or an established cavitated enamel lesion (ICDAS 3) in a clinical examination was considered the yield from the bitewing radiographs. This additional diagnostic yield of radiography was measured on a tooth and on a surface level as well as on an individual level. The yield was calculated as the percentage of surfaces with radiographically detected established or severe dentinal caries (ICDAS 4–6) of all surfaces recorded as ICDAS 0–2 (yield 1) or of surfaces recorded as ICDAS 3 (yield 2), as well as the sum of these two (yields 1 and 2). The number of surfaces with gain was calculated individually.

### 2.5. Calibration and Interexaminer Agreement

In an attempt to estimate the reliability of the radiographic examination, calibration by assessing several pairs of radiographs with a specialist experienced in the radiological analysis of caries (KP) was carried out first. After that, the authors (AHL and KP) independently examined 73 randomlyselected pairs of bitewing radiographs. The assessment of 1 424 surfaces out of 1 435 surfaces examined was agreed upon (99.2% interexaminer agreement rate). The number of established or severe dentine caries lesions agreed upon was 28. There was established or severe dentinal decay in 11 out of these 73 subjects examined. The number of these lesions within a subject varied between zero and nine.

### 2.6. Data Analysis/Statistical Methods

The data input of the clinical and the radiographic registrations was performed by the examining dentist (AHL). Crosstabulation was used for the description of the yield on a tooth surface and on an individual level. The independent samples nonparametric Mann-Whitney *U* test was used to test the occurrence of yield between clinically DMFS = 0 and DMFS > 0 subjects. The level of significance was set to 0.05. The software used for analyzing the data was PASW Statistics Data Editor Software for Windows version 18 (SPSS Inc., Chicago, IL, USA).

### 2.7. Ethics

The parents of the subjects were informed, and they gave written consent before the start of the 4-year clinical trial. It was made clear to each subject that attending the study was fully voluntary. The study was independently reviewed and approved by the Ethics Board of the Kotka Health Center. The analysis here used data collected in the clinical trial. No other data from any other record was combined.

## 3. Results

Forty-four percent of the subjects (*n* = 363) had DMFS = 0 in the clinical examination. The number and the percentage of the surfaces with the different caries categories (established cavitated enamel decay, established and severe dentinal decay) and the filled and excluded surfaces recorded in clinical examination, by tooth and surface, are given in Table 1.

On a tooth surface level, the contribution of the bitewing radiographs was the greatest on the occlusal surfaces of the first molars where established and severe dentinal decay (ICDAS 4–6) was registered in the radiographic examination on forty percent of the established cavitated enamel lesions (ICDAS 3) and on eleven percent of the surfaces registered ICDAS 0–2 clinically. On the approximal surfaces, the yield from the radiographs was the greatest on the mesial surfaces of the first molars (Table 2).

The proportion of the subjects who had more established and severe dentinal decay detected in the radiographs than in the clinical examination, that is, the yield on an individual level, is given in Table 3. Nearly fifty percent of the clinically examined DMFS = 0 subjects benefited from the radiographs, in comparison with the DMFS > 0 subjects, where in nearly sixty percent of the subjects an extra diagnostic BW yield was obtained (yield one: *P* = .005, yield two: *P* = .005, and yields one and two: *P* = .004) (Table 3).

The distribution of subjects according to the sum of the yield surfaces in relation to the clinical findings is given in Table 4. Of subjects with an additional diagnostic yield from the radiographs, most subjects received one yield; in other words, one additional established or sever dentinal lesion was detected in the radiographs. This was true for all categories of yield: one, two, and the combination of these two.

## 4. Discussion

The findings of the present cross-sectional study in 14-year olds belonging to a low-caries population and with practically no prior history of BW screening suggest that in the detection of severe dentinal decay both clinically examined DMFS = 0 and DMFS > 0 adolescents benefit from BW. Even though the contribution of BW to caries diagnosis seemed to be more significant in subjects with earlier dentinal caries experience than in those without, it is noteworthy that in almost fifty percent of subjects regarded as having no need for restorative treatment in a clinical examination additional diagnostic yield was obtained. On a tooth surface level, most of the yield of BW was obtained on the occlusal surfaces of the first and the second molars, where caries was detected in eleven and nine percent, respectively, of the clinically registered ICDAS 0–2 occlusal surfaces.

The clinical relevance of the present study is related to the decision regarding the restorative treatment. The definition of the yield was set on a level in which restorative treatment could not be avoided even if the attitude towards restorative caries treatment had been prudent, in general. Thus, the results of the study show how much severe caries calling for restorative treatment was detected in the radiographs on surfaces which would not have been filled if the decision had been based on the findings of the clinical examination, only.

The WHO criterion used in obtaining the data is not able to identify the subtle features essential in the current understanding of caries progress. Some information has thus been lost when scoring caries using this method. For example, scores zero to two in ICDAS fall below the used threshold for caries. On the other hand, since the ICDAS code 3 was separate, no distortion regarding established enamel decay was caused by the merging of the classes 0 to 2. Albeit obsolete, WHO criteria can be considered compatible with those of ICDAS [24, 25], which made the recoding possible. A rationale behind this transformation from one classification to another in the present study was to bring the data up to date. Braga et al. [24] and Iranzo-Cortés et al. [25] found the two systems comparable when the cutoff point was set at ICDAS 3. The differences between these assessment systems reflect a change in the caries process. In earlier days dental decay penetrated faster to dentine and open dentine cavities were more frequent than today. Thus, there was no need for such accurate assessment systems with several classes focusing merely on enamel decay.

In general, the present findings are in line with those in the earlier studies. On the other hand, one needs to be cautious in making comparisons between studies with different methods of calculating the additional value and variation in the diagnostic threshold and the prevalence of the caries, as well as in the age of the study subjects. Another point is that in the Swedish systematic review [1], the majority of the studies on the value of the BW were *in vitro* studies, the results of which may not directly be comparable with and applicable to the clinical conditions. In some cases, the diagnostic threshold may not have been specified, as observed in two other reviews [10, 26]. Many of the earlier studies have been made in conditions with high-caries prevalence [12], with the subjects either younger [12, 27] or older [28], or they have reported the additive effect of the BW on the approximal surfaces only [3, 26, 27].

The present finding in which 53% of the sample subjects benefited from the radiographs is consistent with that of Lillehagen, who found that 48% of 9-year olds, likewise belonging to a low-caries prevalence population, benefited from BW screening [27]. Our finding of this high proportion of the clinically DMFS = 0 subjects with a yield from BW is also supported by that of Mestriner et al. [5] and may indicate that in adolescents belonging to a low-caries prevalence population the absence of caries in a clinical examination may not be an adequate criterion when considering the need for obtaining the first pair of bitewing radiographs.

Regarding the occlusal surfaces, our finding also agrees with those of Weerheijm et al. [7, 15, 29] and Hintze [30], who have reported on the considerable diagnostic yield provided by BW screening in the detection of hidden occlusal caries in adolescents. Similar yield on the occlusal surfaces has also been reported by Sawle and Andlaw [6] and Hopcraft and Morgan [28]. In the present 14-year olds, the yield in the second molars was surprisingly similar to that of the first molars. Thus, it seems that in this sample the occlusal caries lesions of the second molars progress at a clearly faster rate than in the first molars.

Even though the visual/tactile examination is not considered sufficient in the detection of dentine caries on the approximal surfaces of the posterior teeth, with a sensitivity of 40 percent [1], and BW has been recommended in the examination of approximal surfaces of adolescents older than 12 years [31], in the present study the clinically undetected deep caries was diagnosed on only three percent of the clinically healthy approximal surfaces of the first molars. This yield may be somewhat smaller than in some other studies [12, 28]. Since these two other studies were conducted either in high-caries prevalence conditions [12] or with subjects older than in the present trial [28], the results cannot be directly compared with those in the present one. On the other hand, it is worth mentioning that on nearly twenty percent of the approximal surfaces of the first molars clinically diagnosed as established enamel decay, established or severe dentine decay was detected in the BW. However, the prevalence of the approximal caries was altogether low in this sample of relatively young adolescents, and one has to be cautious in drawing conclusions from this finding.

In our study, the diagnostic threshold was set to the obvious dentine caries level. Because of the anatomical relations, the ability of BW to detect enamel as well as early dentine lesions on the occlusal surfaces can be considered poor. It was also thought that the presumably high number of border-line diagnoses between the enamel and the dentine lesions might have a confounding effect on the result, thus advocating the diagnostic threshold chosen. Likewise, it has to be born in mind that, with decreasing prevalence of the disease, the proportion of false-positive registrations in dentine increases significantly [1]. We also wished to discover whether in a low-caries prevalence population a judgment regarding the need for BW screening can be reliably made on the basis of the clinical findings.

As mentioned earlier, the majority of the first molars, which had erupted seven to eight years earlier, were now examined radiographically for the first time. Likewise, it needs to be recalled that most of the first molars were covered with a fissure sealant, which may make it more difficult to establish the presence of occlusal caries in a case of a possible leakage, as noted in the studies of Weerheijm et al. [7, 15]. Thus, the relatively high number of hidden established and severe dentine lesions detected in the molars of the present study may to some extent be related to these conditions of this particular study. The prevalence of hidden caries may also be pronounced because of the relatively high level of natural fluoride some subjects had been exposed to in their childhood. Likewise, the use of fluoride dentifrice was regular and the use of topical fluoride applications had earlier been frequent among the subjects. As a result, the external validity of our findings may be affected and the findings may be generalized to low-caries populations only.

In addition, in the low-caries conditions, the rate of the progression and morphology of the lesions differ from those in the high-prevalence conditions, making the clinical detection of the lesions more challenging. In the present sample, the rate of caries progression was relatively slow with regard to the first molars, which had erupted seven to eight years ago, and no lesions calling for endodontic treatment were observed, for example. On the other hand, the frequency of the hidden established and severe lesions in the second molars had nearly reached that of the first molars even though they had erupted not more than one to three years before the BW.

The data was collected on a tooth surface level in well-equipped dental clinics, which can be considered the strength of the study. We would nonetheless like to consider some possible weaknesses and limitations of the study. Due to practical reasons, the clinical examinations were performed by only one calibrated examining dentist. That was also the case in the studies of Hopcraft and Morgan [28] and Lillehagen et al. [27]. Another confounding factor could be that the intraexaminer variation was not tested. However, these eventual shortcomings could be at least in part compensated for by the independent double-readings of 20% of the radiographs by an experienced calibrated dentist.

One limitation regards the method as such; the use of only one radiographic measurement point precludes the possibility of distinguishing between an active and an arrested lesion [32]. The impression of the size of the additional value of BW screening and of one's likelihood of developing more caries may be skewed because of lack of knowledge concerning the prior history of caries in each subject examined, the tooth colored restorations, and a difficulty in discerning a difference between sealants and fillings.

Further, due to these limitations of the clinical examination, as well as because of being the first radiographic examination for most of the subjects, the finding of the present cross-sectional study may actually exaggerate the size of the contribution of BW screening and thus not be directly applicable to everyday clinical practice, where the relationship with the patient is of a different character.

Bitewing examination is an important tool in the detection of caries in everyday clinical practice. However, there is a large variation in the frequency of its use between countries; thus, the eventual recommendations on the frequency and indications of its use need to be reflected upon those existing in that particular country at issue. It is also worthy of remark that, even though BW screening is used more or less routinely in the annual clinical examinations in Sweden and Norway, there seems to be no difference in the improvement of dental health in relation to Nordic countries with a more restricted attitude in obtaining the radiographs [1]. In a low-caries prevalence population exposed to an optimal amount of fluorides, the interval between radiographic examinations can be longer than one year without having a negative effect on one's dental health [33], and the need for radiographs should be based on a comprehensive individual risk estimation [1].

Earlier, when caries was relatively easy to detect even with the naked eye, the criterion for obtaining the radiographs if caries is detected in a clinical examination may have been a meaningful one. This is also the recommendation of the evidence-based Current Care Guidelines in Finland [2]. It is, however, known that there are occasions where the enamel appears intact, but there is a pronounced spread of caries into dentine. In other words, hidden caries lesions do occur [1, 30]. The present findings suggest that the absence of dentinal caries in a clinical examination may not be a sufficient criterion in assessing the need for BW. Rather, obtaining radiographs should be considered at the latest at this point, from subjects with no prior bitewing examination, in particular. This practice could serve as a safety net, aiming at avoidance of pain and endodontic treatment.

Further research in this area might produce material for clinicians regarding the need for BW screening in different age groups. Likewise, it is a well-known phenomenon that the diagnostics of caries vary from one clinician to another. This leads to variation in the treatment between the patients. Thus, there is a clear need for standardization and further research is called for [1].

## 5. Conclusions

It is concluded that in the low-caries prevalence population a remarkable portion of both clinically DMFS = 0 and DMFS > 0 14-year olds may benefit from BW screening. Most of the benefit of BW is obtained on the occlusal surfaces of the first permanent molars and nearly as frequently on the occlusal surfaces of the second permanent molars.

## Figures and Tables

**Table 1:** Number and percentage of surfaces with different stages of decay in clinical examination by tooth and surface.

	ICDAS 0–2^a^	ICDAS 3^b^	ICDAS 4–6^c^	Filled	Surfaces excluded
	*n* (%)	*n* (%)	*n* (%)	*n* (%)	*n* (%)
*Maxilla *					
1st premolar					
Distal	662 (91.2)	10 (1.4)	2 (0.3)	1 (0.1)	51 (7.0)
2nd premolar					
Mesial	687 (94.6)	6 (0.8)	0 (0.0)	0 (0.0)	33 (4.5)
Distal	678 (93.4)	0 (0.0)	0 (0.0)	1 (0.1)	47 (6.5)
1st molar					
Occlusal	575 (79.2)	46 (6.3)	30 (4.1)	69 (9.5)	6 (0.8)
Mesial	617 (85.0)	59 (8.1)	9 (1.2)	15 (2.1)	26 (3.6)
Distal	691 (95.2)	3 (0.4)	0 (0.0)	5 (0.7)	27 (3.7)
2nd molar					
Occlusal	590 (81.2)	57 (7.9)	32 (4.4)	13 (1.8)	34 (4.7)
Mesial	689 (94.9)	2 (0.3)	1 (0.1)	0 (0.0)	34 (4.7)
*Mandible *					
1st premolar					
Distal	710 (97.8)	0 (0.0)	0 (0.0)	0 (0.0)	16 (2.2)
2nd premolar					
Mesial	678 (93.3)	0 (0.0)	0 (0.0)	1 (0.1)	47 (6.5)
Distal	670 (92.2)	8 (1.1)	1 (0.1)	0 (0.0)	47 (6.5)
1st molar					
Occlusal	507 (69.8)	68 (9.4)	48 (6.6)	97 (13.4)	6 (0.8)
Mesial	636 (87.6)	55 (7.6)	10 (1.4)	14 (1.9)	11 (1.5)
Distal	705 (97.1)	1 (0.1)	1 (0.1)	7 (1.0)	12 (1.7)
2nd molar					
Occlusal	545 (75.1)	67 (9.2)	39 (5.4)	52 (7.2)	23 (3.2)
Mesial	690 (95.0)	12 (1.7)	0 (0.0)	2 (0.3)	22 (3.0)

Sum of surfaces in each row = 726.

Total number of surfaces analyzed = 11 616.

^a^WHO 0, 1.

^b^Established enamel lesion with a clinically detectable loss of substance.

^c^Established and severe dentinal decay calling for filling.

**Table 2:** Number of ICDAS 0–2 surfaces in both clinical examination and with BW included and number of surfaces with yield from radiographs.

	ICDAS 0–2^a^ in both clinical exam and BW	ICDAS 0–2^a^ in clinical exam, ICDAS 4–6^c^ in BW (yield 1)	ICDAS 3^b^ in clinical exam, ICDAS 4–6^c^ in BW (yield 2)
	*n* (%)^1^	*n* (%)^1^	*n* (%)^2^
1st premolar			
Distal	1324 (96.5)	12 (0.9)	0 (0.0)
2nd premolar			
Mesial	1324 (97.0)	5 (0.4)	0 (0.0)
Distal	1269 (93.3)	18 (1.3)	0 (0.0)
1st molar			
Occlusal	963 (89.0)	114 (10.5)	45 (39.5)
Mesial	1110 (88.6)	40 (3.2)	22 (19.3)
Distal	1308 (93.7)	11 (0.8)	0 (0.0)
2nd molar			
Occlusal	1032 (90.9)	98 (8.6)	42 (33.9)
Mesial	1324 (96.0)	3 (0.2)	0 (0.0)

^1^% of surfaces registered clinically ICDAS 0–2.

^2^% of surfaces registered clinically ICDAS 3.

^a^ICDAS 0–2, WHO 0, 1.

^b^ICDAS 3, enamel lesion with a clinically detectable loss of substance.

^c^Established or severe dentine decay = ICDAS 4–6, dentine caries obviously spreading in the dentine.

**Table 3:** The number and proportion of subjects with yield from the radiographic examination in relation to the clinical findings.

Subjects	*N *	Yield 1	Yield 2	Yields 1 and 2
		*n* (%)	*n* (%)	*n* (%)
DMFS = 0	161	76 (47.2)	25 (15.5)	77 (47.8)
DMFS > 0	202	117 (57.9)	57 (28.2)	119 (58.9)

**Table 4:** The distribution of subjects according to the sum of yield surfaces in relation to the clinical findings.

Subjects	*N *	Yields per subject	Yield 1*	Yield 2**	Yields 1 and 2***
			*n* (%)	*n* (%)	*n* (%)
DMFS = 0^a^	161	0	85 (52.8)	136 (84.5)	84 (52.2)
		1	44 (27.3)	18 (11.2)	39 (24.2)
		2	20 (12.4)	6 (3.7)	14 (8.7)
		3	5 (3.1)	1 (0.6)	10 (6.2)
		4	4 (2.5)	—	8 (5.0)
		≥5	1 (0.6)	—	6 (3.7)

DMFS > 0^b^	202	0	85 (42.1)	145 (71.8)	83 (41.1)
		1	47 (23.3)	44 (21.8)	39 (19.3)
		2	36 (17.8)	8 (4.0)	27 (13.4)
		3	18 (8.9)	5 (2.5)	22 (10.9)
		4	8 (4.0)	—	9 (4.5)
		≥5	8 (4.0)	—	22 (10.9)

Stat. Sign. independent samples Mann-Whitney *U* test nonparametric.

*A statistically significant difference in the distribution of yield one between DMFS = 0 and DMFS > 0 subjects *P* = .005.

**A statistically significant difference in the distribution of yield two between DMFS = 0 and DMFS > 0 subjects *P* = .005.

***A statistically significant difference in the distribution of yields one and two between DMFS = 0 and DMFS > 0 subjects *P* = .004.
